# Early sexual initiation and risky sexual practices among alcohol- and tobacco-using young adults in Taiwan: mediation analysis of preceding-sex use of illicit drugs

**DOI:** 10.1186/s12889-020-09777-0

**Published:** 2020-11-03

**Authors:** Tat Leong Wu, Te-Tien Ting, Chuan-Yu Chen, Lien-Wen Su, Wei J. Chen

**Affiliations:** 1 Institute of Epidemiology and Preventive Medicine, College of Public Health, National Taiwan University, Taipei, Taiwan; 2 School of Big Data Management, Soochow University, Taipei, Taiwan; 3 Institute of Public Health, National Yang-Ming University, Taipei, Taiwan; 4 Center for Neuropsychiatric Research, National Health Research Institutes, Zhunan, Miaoli County Taiwan; 5 Kunming Prevention and Control Center, Taipei City Hospital, Taipei, Taiwan; 6 Department of Public Health, College of Public Health, National Taiwan University, Taipei, Taiwan; 7 Department of Psychiatry, National Taiwan University Hospital and College of Medicine, National Taiwan University, Taipei, Taiwan

**Keywords:** Early sexual initiation, Risky sexual practices, Preceding-sex use of illicit drugs, Substance use, Sexual health, Respondent-driven sampling

## Abstract

**Background:**

As early sexual initiation is increasingly common in East Asia, we examined its relations to risky sexual practices in alcohol- and tobacco-using individuals in Taiwan and evaluated whether the associations were mediated through preceding-sex use of illicit drugs.

**Methods:**

Participants, recruited from alcohol- and tobacco-using adults aged 18 to 50 in Taipei through respondent-driven sampling (*N* = 1115), completed a computer-assisted self-interview covering questions on substance use and sexual experiences. In a subsample of 916 participants who had had sexual experience (median age 27), we examined the relations of early sexual initiation (< 16 years) to multiple sexual partners, casual sex, group sex, and rare condom use. Causal mediation analyses were conducted to examine whether illicit drug use preceding sex mediated these associations.

**Results:**

Around 9.3% reported early sexual initiation and the prevalence of risky sexual practices ranged from 7% (group sex) to 47% (rare condom use). Early initiators had a higher prevalence of regular binge drinking, illicit drug use, and risky sexual practices. In the multivariable analyses, higher odds of multiple sexual partners, casual sex, and group sex were consistently associated with early sexual initiation, gender, and their interaction. Mediation through preceding-sex use of illicit drugs was found between early sexual initiation and the three risky sexual practices, with the proportions mediated ranging from 17 to 19%.

**Conclusions:**

Early sexual initiators were more likely to engage in risky sexual practices and preceding-sex use of illicit drugs partially explained this relationship, calling for more attention to this population’s sexual health.

**Supplementary Information:**

The online version contains supplementary material available at 10.1186/s12889-020-09777-0.

## Background

Early sexual initiation [1, 2] has been associated with various risky sexual practices, including multiple sexual partners, sex with nonregular partners, sex under the influence of alcohol or drugs, and condom nonuse [3–7]. Despite not being uniformly defined, having sex before age 16 has been commonly defined as early sexual initiation by many studies in Europe, the US, and Taiwan [1, 2, 8, 9]. This relationship might be moderated by gender, since males were found to hold more permissive sexual attitudes than females [10, 11]. In East Asia, where collectivist culture is a common tradition, early sexual behaviors tend to be disapproved [12, 13]. For example, in Taiwan, only 3% male and 1.6% female 12th graders (approximately 18 years old) nationwide, respectively, were early sexual initiators [2]. But in the UK, around one-third of its people aged 16–24 nationwide were early initiators [14]. Since traditional culture might have been weakened owing to widespread education and increased exchange with Western societies, there have been signs that early sexual initiation is slowly becoming more common in East Asia [2, 15–18].

Risky sexual behavior has been correlated with use of a variety of substances among young people. In particular, illicit drug use among adolescents would increase the odds of risky sexual behavior further than alcohol or tobacco use [19, 20]. A meta-analysis found the overall effect size for the relationship between substance use (alcohol, marijuana, hard drugs, drugs and alcohol) and risky sexual behavior was in the small to moderate range, except a large effect size for multiple substance use and multiple risky sexual behavior [21]. Furthermore, the relationship between illicit drug use and risky sexual practices is complicated as it could also be moderated by gender [21].

Previous studies have found that young people who had preceding-sex (i.e., right-before-sex) use of illicit drugs might have intentions for increasing the likelihood of sex, condom nonuse, or sexual enhancement [22–25]. The emergence of so-called club drugs [26] or party drugs [27] has led to a change in the landscape of illicit drug use, especially among young people. In Taiwan, the pattern of illicit drug use underwent drastic changes in 1990s, starting with methamphetamine’s surge during this period to the upgrading of ecstasy to Schedule II controlled drugs in 1999 [28]. An increasing popularity of ketamine, ecstasy, and marijuana has been consistently found among school-attending adolescents in national surveys from 2004 to 2006 [29], as well as among alcohol- and tobacco-using young adults recruited via social network from 2007 to 2010 [30, 31]. The most recent 2014 national survey in Taiwan found that the prevalence estimates of club (or party) drugs (mainly ketamine, marijuana, and ecstasy) were slightly higher than hard drugs (mainly methamphetamine and heroin), and that club drug users were different from hard drug users in socio-behavioral correlates and psychosocial distress [32]. With the increasing prevalence of club drug use in Taiwan, it is worthwhile to examine whether the likelihood of risky sexual practices under the influence of these illicit drugs [33] was increased among its young population.

Besides risky sexual practices, substance use was also shown to be associated with early sexual initiation. Evidence from cross-country studies showed that substance use (measured by tobacco use frequency, alcohol use frequency, and ever drunk) was consistently associated with increased odds of early sexual initiation [1, 34]. Earlier data in the US have pointed out that early-onset of marijuana or other illicit drugs were stronger predictors for having intercourse initiation before age 16 [35], although a study in East Asia indicated that the majority of club-drug users had sexual initiation before their first club-drug use [6]. The association of early initiation of illicit drug use with early initiation of sexual behavior might be attributed to shared underlying pathways, such as certain personality traits or disadvantaged environment [6]. For example, sensation seeking and impulsivity have linked with a wide array of underage risk behaviors, including substance use [36–39] and early sexual initiation [40–45]. Also, the observed association may reflect a causal pathway. Specifically, as drug initiation emerges one’s peer or social networks may evolve and later increase the exposure to more drug-experienced adults and the engagement in adult-oriented activities, including sexual behaviors [46].

Hence*,* the associations between early sexual initiation and risky sexual practices might be mediated via the influence of drugs [21]. Previous studies found that early sexual initiators were more likely to report preceding-sex use of illicit drugs [6, 7]. Meanwhile, studies found that preceding-sex use of illicit drugs was associated with risky sexual practices [47, 48]. Given that illicit drug users reported the use of illicit drugs to enhance sensation and to reduce inhibition [24], it is worthwhile to examine whether preceding-sex use of illicit drugs acted as a mediator between early sexual initiation and risky sexual practices. In particular, the recent development of causal mediation analysis [49, 50] extends the classical mediation approach [51] to provide additional flexibility to address this issue.

A challenge in studying early sexual initiation and illicit drug use in East Asian populations is the relatively low prevalence of illicit drug use in the general population. To address these gaps, we turned to a sample of alcohol- and tobacco-using young adults in Taiwan [30] that was recruited via respondent-driven sampling (RDS) [52]. In this study, early sexual initiation was defined as having sexual experience before age 16, which is the age definition used in another study of early sexual initiation in Taiwan [2]. Based on this RDS sample, this study aims to (1) examine the associations between early sexual initiation and four risky sexual practices (multiple sexual partners, casual sex, group sex, and rare condom use), meanwhile testing the moderation of gender on early sexual initiation; and (2) explore whether the associations between early sexual initiation and each of the risky sexual practices were mediated through preceding-sex use of illicit drugs using a causal mediation analysis.

## Methods

### Participants

We utilized a sample of alcohol- and tobacco-using young adults recruited using RDS in the Taipei metropolitan area from 2007 to 2010, which was described in more detail elsewhere [30]. Briefly, two sampling criteria were set for the seeds and the recruits of this RDS recruitment: (a) adult residents living in the Taipei metropolitan area, aged 18 to 50 years; and (b) regular alcohol and tobacco users. We used a higher age cap of 50 to facilitate recruitment for the participants. The study recruited 47 seeds from either community-based or hospital-based settings. Seeds and eligible participants could invite up to six peers who satisfied the sampling criteria to participate in the study.

Participants were informed of the nature of the study and were guaranteed confidentiality right before the survey. The total sample size was 1115 (median age = 26; interquartile range: 22–32), among whom 919 were sexually experienced and 916 of them (median age = 27; interquartile range: 23–33) provided information on their age at first sex. Although our analyses were limited to the 916 individuals with sexual initiation data, the original sample of 1115 was kept in order to derive complete referral chains in RDS-specific estimation by denoting those without information on sexual initiation as missing in the outcome variables. The institutional review board of the College of Public Health, National Taiwan University, approved this study. More information about the recruitment of seeds, the balance of referral and confidentiality, and participants’ alcohol and tobacco use is provided in Additional file 1: Supplementary Methods.

### Measures

Participants completed the survey through an audio computer-assisted self-interview (ACASI) implemented on notebook computers. The questionnaire comprised sections on social demographic characteristics, sexual experiences, and substance/illicit drug use, among others. Additional file 2 provides the questionnaire for the variables used in this study.

#### Sexual experience

We defined sexually experienced as having experience in any of the following three forms: vaginal sex, oral sex, or anal sex. Since various types of sexually transmitted infections could be spread through oral sex, we counted it as sexual experience. We categorized a respondent as an early sexual initiator if the age reported for any one type of sexual behaviors was lower than 16, following a previous study [2]. Four types of risky sexual practices were further defined as binary variables as follows (more details in Additional file 1: Supplementary Methods): (1) multiple sexual partners: having ≥8 sexual partners in lifetime; (2) casual sex: ever having the experience of one-night stand; (3) group sex: ever having sex with two or more people at the same time or in a row; and (4) rare condom use: a condom use frequency of “seldom” or “never”.

For respondents who reported lifetime use of any illicit drugs, they were further questioned about their experience of using such drugs right before sex. We used the information regarding the gender(s) of people with whom the respondent had sex (male, female, both genders) to define homosexual/bisexual experiences.

#### Substance/drug use experience

Inquiry about use of licit substances (tobacco and alcohol) and illicit drugs started by asking participants about their experience of ever use. For each substance/drug endorsed, respondents were asked further substance/drug-specific questions regarding age of first use, situation of first use, average frequency of consumption, and recency of use. For this study, variables on substance/drug use included the following: (1) regular binge drinking: defined as five or more units of alcohol in one setting “almost every time” or “every time”; and (2) illicit drugs, including those commonly reported in previous studies in Taiwan: ketamine, ecstasy, cannabis, methamphetamine, heroin, FM2 (flunitrazepam), angel dust (phencyclidine), and GHB (4-hydroxybutanoic acid). Following Chen et al., [32] illicit drugs were categorized as (a) hard drugs, including heroin and methamphetamine, which are classified as Schedule I and II controlled drugs, respectively, in Taiwan, or (b) club drugs, including ecstasy, ketamine, cannabis, FM2, phencyclidine, and GHB, which are classified as Schedule III controlled drugs in Taiwan except cannabis and ecstasy (classified as Schedule II).

### RDS-based prevalence estimation

We computed RDS-weighted prevalence estimates using the RDS Analysis Tool (RDSAT) version 7.1 [53]. Confidence intervals (CIs) of the estimated population prevalence were obtained by bootstrapping the RDS sample. We followed the recommendation of the software manual to increase the number of bootstrap resamples to 15,000 to obtain higher accuracy for the 95% CIs reported in this study. Other estimation options remained default.

### Weighted logistic regression analysis

Using the weights in the RDS network output by RDSAT, as recommended by Wejnert and Heckathorn [54], we built multivariable logistic models to regress each risky sexual practice on early sexual initiation while adjusting for potential confounders. To examine whether gender would moderate early sexual initiation, we included an interaction between gender and early sexual initiation in each of the logistic regression models tested. Logistic regression analyses were done using Stata 14.0 [55].

### Mediation analysis

We carried out a mediation analysis following the formulation by Valeri and VanderWeele [50] with a user-written package that was run in Stata 14.0 [56]. For the outcome regression modeling, following the suggestion by VanderWeele [49], Poisson regression was used for outcomes that were not rare (a prevalence above 10%) and logistic regression for rare outcomes (below 10%). When Poisson regression was used, the direct, indirect, and total effect estimated by the mediation analysis would be interpreted as the risk ratio, rather than the odds ratio when logistic regression was used. In addition, we did not allow for interaction between exposure and mediating variables as their interaction was nonsignificant in all four outcome regression models. Without the interaction term, the direct effect need not be differentiated into the controlled direct effect and natural direct effect [50]. A total of 1000 bootstraps were implemented to obtain 95% confidence intervals in this study.

## Results

Out of 916 sexually experienced participants, 109 (a weighted prevalence of 9.3, 95% CI: 6.9–12.1) were early sexual initiators. The proportion of early sexual initiators among males (79 out of 578, 11.0, 95% CI: 7.8–14.8) was higher than that among females (30 out of 338, 6.9, 95% CI: 3.7–11.3).

As displayed in Table 1, participants with early sexual initiation had a tendency of higher proportion of males (68.3%) than those with nonearly sexual initiation (56.3%; *p* = 0.085). Hence, the remainder of Table 1 is stratified by gender. Male early sexual initiators had lower educational attainment and a higher proportion of being unemployed, or having a part-time job or in the military than male nonearly sexual initiators. Meanwhile, female early sexual initiators had lower educational attainment and a higher proportion of living with marital or romantic partner than female nonearly sexual initiators.

**Table 1:** Demographic characteristics of sexually experienced alcohol- and tobacco-using young adults in Taipei metropolitan area recruited using RDS during 2007–2010, by gender and sexual initiation (*N* = 916)

	Nonearly sexual initiation			Early sexual initiation			
Variable	*N*	%_wt_	95% CI	*N*	%_wt_	95% CI	*p*^a^
*Total*	*N* = 807			*N* = 109			
Gender							.085
Male	499	56.3	(50.2–63.0)	79	68.3	(54.2–81.4)	
Female	308	43.7	(36.9–49.7)	30	31.7	(18.6–45.9)	
Age (in years), median (IQR)^b^	807	27.0	(23.0–33.0)	109	25.0	(21.0–30.0)	.081
*Males*	*N* = 499			*N* = 79			
Age (in years), median (IQR)^b^	499	26.0	(23.0–33.0)	79	26.0	(22.0–32.0)	.630
Education level < college	291	59.9	(52.2–67.1)	62	83.5	(73.2–92.5)	.004**
Employment							.002**
Full-time job	294	31.7	(24.5–38.6)	39	27.4	(12.5–45.5)	
Work-study/in school	153	54.8	(47.2–62.7)	16	37.5	(22.5–52.3)	
Unemployed/part-time job/military	52	13.5	(8.9–18.9)	24	35.1	(20.9–50.3)	
Living with marital or romantic partner	89	16.6	(11.9–21.5)	14	13.4	(4.4–24.0)	.548
*Females*	*N* = 308			*N* = 30			
Age (in years), median (IQR)^b^	308	28.0	(23.0–34.0)	30	22.5	(20.0–26.0)	.004**
Education level < college	202	75.5	(67.4–81.9)	25	88.6	(72.7–99.1)	.034*
Employment							.009**
Full-time job	204	15.8	(9.8–22.2)	17	44.5	(16.7–71.8)	
Work-study/in school	72	68.1	(55.5–75.6)	10	49.7	(24.9–79.1)	
Unemployed/part-time job/military	32	16.1	(9.7–29.1)	3	5.8	(0–17.6)	
Living with marital or romantic partner	96	21.1	(16.0–28.9)	13	71.0	(48.4–89.0)	<.001***

^a^Using weighted chi-square tests, with weight exported from RDSAT; *p < 0.05, ** *p* < 0.01,*** *p* < 0.001

^b^This estimate was unweighted due to a limitation of RDSAT. Its corresponding group comparison *p*-value was generated from rank-sum test

The two groups of sexual initiation were compared in the experience of regular binge drinking and a variety of illicit drug use experiences (Table 2). For males, early sexual initiators were more likely to report regular binge drinking and to have ever used illicit drugs, with ketamine being the most commonly used (41.6% vs. 12.1%), as well as have the experience of preceding-sex use of illicit drugs than nonearly sexual initiators. For females, early sexual initiators were more likely to have ever used ketamine (37.9% vs. 11.8%). Nevertheless, the small sample size of the female early sexual initiation group (*n* = 30) did not allow for meaningful comparisons for several illicit drugs variables and their preceding-sex use of illicit drugs.

**Table 2:** Substance use among sexually experienced alcohol- and tobacco-using young adults in Taipei metropolitan area recruited using RDS during 2007–2010, by gender and sexual initiation (*N* = 916)

	Nonearly sexual initiation			Early sexual initiation			
Variable	*N*	%_wt_	95% CI	*N*	%_wt_	95% CI	*p* ^a^
*Males*	*N* = 499			*N* = 79			
Regular binge drinking^b^	115	20.5	(15.9–26.3)	31	35.2	(21.0–49.2)	.020*
Illicit drug use (lifetime)							
Any illicit drug	132	27.5	(21.3–36.2)	46	53.7	(38.2–71.0)	<.001*
Ketamine	70	12.1	(8.6–16.9)	34	41.6	(27.5–58.7)	<.001***
Ecstasy	68	13.0	(9.1–18.2)	22	25.1	(13.4–40.6)	.020*
Cannabis	74	11.5	(7.8–16.5)	23	28.3	(16.2–43.9)	<.001***
FM2/angel dust/GHB	14	2.2	(0.3–4.7)	11	11.8	(3.9–22.4)	<.001***
Methamphetamine	39	6.6	(3.4–10.1)	20	24.9	(12.0–38.8)	<.001***
Heroin	20	3.2	(0.7–6.4)	12	10.0	(1.7–19.4)	.007**
Hard drug use^c^	41	7.3	(3.7–11.1)	22	26.9	(13.0–40.7)	<.001***
Exclusively club drug use^d^	75	14.8	(10.5–20.2)	21	25.0	(13.3–39.2)	.079
Illicit drug use right before sex	42	6.7	(3.4–10.1)	21	27.5	(15.1–43.2)	<.001***
*Females*	*N* = 308			*N* = 30			
Regular binge drinking^b^	46	11.1	(6.4–16.4)	8	18.3	(0.7–38.4)	.342
Illicit drug use (lifetime)							
Any illicit drug	70	18.9	(12.6–26.6)	13	38.8	(15.2–65.0)	.064
Ketamine	41	11.8	(6.9–17.7)	11	37.9	(15.0–67.0)	.005**
Ecstasy	39	12.5	(7.1–18.8)	7	14.7	(2.3–33.4)	.750
Cannabis	45	12.0	(7.2–18.3)	3	7.4	(0.0–21.0)	.492
FM2/angel dust/GHB	4	1.2	(0.0–3.0)	0	0.0	–	.677
Methamphetamine	13	5.8	(1.8–11.1)	2	5.0	(0.0–16.0)	.874
Heroin	8	3.3	(0.1–8.5)	0	0.0	–	.492
Hard drug use^c^	15	6.5	(1.6–13.2)	2	5.0	(0.0–16.3)	.755
Exclusively club drug use^d^	30	5.3	(3.0–8.8)	6	20.0	(1.4–46.7)	.055
Illicit drug use right before sex	34	11.6	(6.5–18.1)	5	14.1	(1.5–30.7)	.751

^a^ Using weighted chi-square tests, with weight exported from RDSAT; *p < 0.05, ** *p* < 0.01,*** *p* < 0.001

^b^ Five or more units of alcohol in one setting “almost every time” or “every time”

^c^ Hard drugs: methamphetamine and heroin

^d^ Club drugs: ketamine, ecstasy, cannabis, and FM2/angel dust/GHB

Table 3 compares the sexual experience of the two groups of sexual initiation. For males, early sexual initiators were more likely to have one risky practice (group sex) than nonearly sexual initiators, with borderline increase in two risky sexual practice. For females, early sexual initiators were more likely to have all four risky sexual practices (i.e., multiple sexual partners, casual sex, group sex, and rare condom use) than nonearly sexual initiators, but they were not more likely to have had homosexual/bisexual experiences.

**Table 3:** Sexual history among alcohol- and tobacco-using young adults in Taipei metropolitan area recruited using RDS during 2007–2010, by gender and sexual initiation (*N* = 916)

	Nonearly sexual initiation			Early sexual initiation			
Variable	*N*	%_wt_	95% CI	*N*	%_wt_	95% CI	*p*^a^
*Males*	*N* = 499			*N* = 79			
No. of sexual partners							.056
1–3	249	47.4	(40.2–53.4)	24	29.4	(15.5–42.9)	
4–7	120	26.7	(21.2–33.3)	21	31.6	(17.6–47.6)	
≥ 8	130	25.9	(20.4–32.3)	34	39.0	(24.8–54.8)	
Casual sex	206	44.7	(38.1–51.1)	42	53.4	(39.0–69.7)	.267
Group sex	42	7.0	(4.1–10.6)	16	20.9	(10.1–33.6)	.001**
Rare condom use^b^	92	20.6	(15.1–25.8)	21	27.9	(15.3–41.9)	.285
Homosexual/bisexual experience	47	5.4	(2.8–9.1)	12	11.5	(2.0–27.1)	.097
*Females*	*N* = 308			*N* = 30			
No. of sexual partners							.001**
1–3	190	65.4	(55.3–71.7)	8	25.8	(5.2–51.9)	
4–7	97	30.0	(24.0–39.5)	13	54.2	(27.0–80.0)	
≥ 8	21	4.6	(2.3–7.9)	9	20.0	(3.9–39.1)	
Casual sex	69	18.7	(13.5–26.0)	17	72.0	(48.8–90.3)	<.001***
Group sex	13	2.9	(1.3–5.2)	4	16.6	(1.0–38.9)	.004**
Rare condom use^b^	92	34.6	(24.3–42.5)	13	64.2	(38.7–83.9)	.015*
Homosexual/bisexual experience	33	8.0	(4.1–12.5)	1	5.1	(0.0–15.8)	.672

^a^ Using weighted chi-square tests, with weight exported from RDSAT; *p < 0.05, ** *p* < 0.01,*** *p* < 0.001

^b^ Including use frequency of “seldom” and “never”

**Fig. 1:**
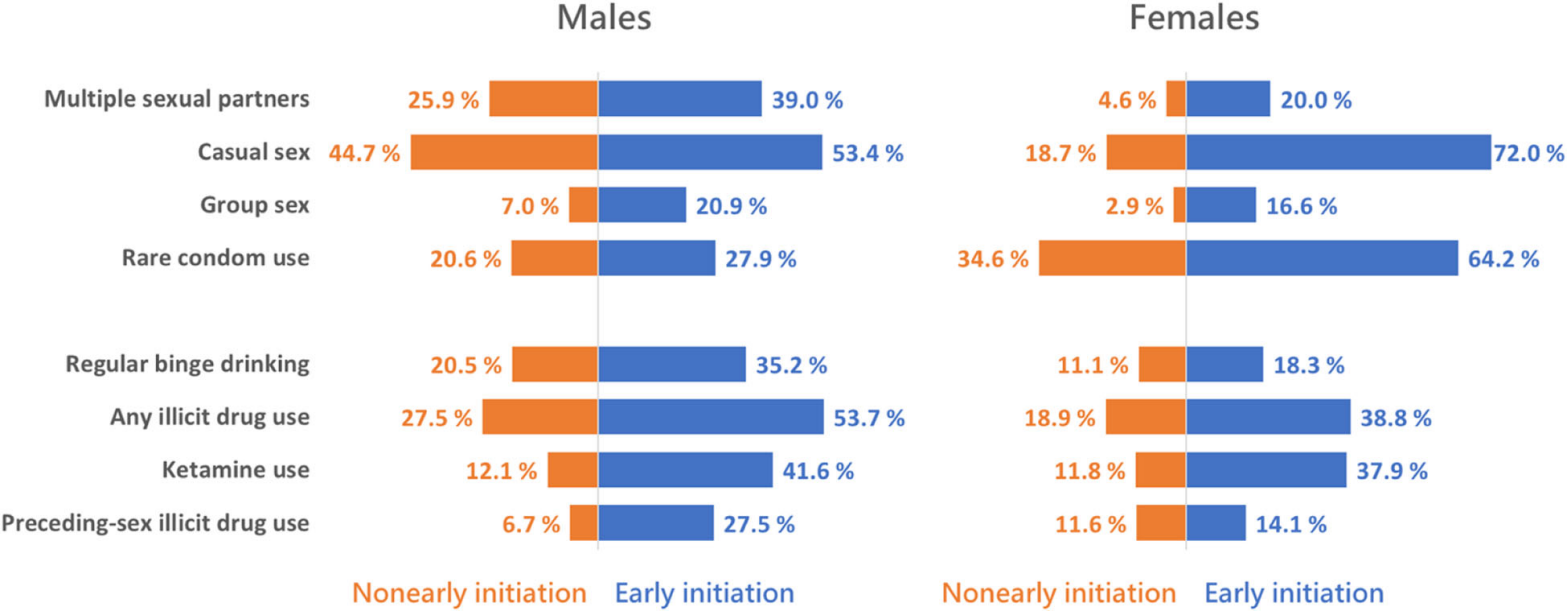
The contrast in the weighted prevalence of four risky sexual practices (multiple sexual partners, casual sex, group sex, and rare condom use) and substance use (regular binge drinking, any illicit drug use, ketamine use, and preceding-sex illicit drug use) between nonearly sexual initiators and early sexual initiators, separately for males and females, among sexually experienced alcohol- and tobacco-using young adults in Taipei metropolitan area recruited using RDS during 2007–2010 (total *N* = 916)

The contrasts in risky sexual practices and substance use between the two groups of sexual initiation stratified by gender are depicted in Fig. 1. The ratios of early initiators to nonearly initiators were greater for females in risky sexual practices and greater for males in substance use, particularly for preceding-sex use of illicit drugs.

The relation of each risky sexual practice to early sexual initiation and gender was evaluated using the weighted multivariable logistic regression with an interaction term in the model to control for potential confounders that were not the mediator of interest in this study, including age, education level, employment, living with marital or romantic partner, and homosexual/bisexual experience (Table 4). Since the interaction term was significant for all risky sexual practices except rare condom use, the corresponding aORs were listed separately for each combination of early sexual initiation and gender. Compared to female nonearly sexual initiators, male nonearly sexual initiators had an aOR of > 1 for multiple sexual partners, casual sex, and group sex, and the magnitude of aOR increased for male early sexual initiators. Regarding multiple sexual partners, we ran a second model to adjust for the years since sexual initiation and the results remained similar. Nevertheless, the relation of rare condom use was associated with early sexual initiation only for females with a borderline significance (*p* = .051). When the number of sexual partners was added as covariate in a second model, reported in Additional file 1: Supplementary Results, rare condom use remained associated with early sexual initiation only for females.

**Table 4:** Weighted multivariable logistic regression models of multiple sexual partners, casual sex, group sex, and rare condom use on early sexual initiation among alcohol- and tobacco-using young adults in Taipei metropolitan area recruited using RDS during 2007–2010 (*N* = 916)

	Multiple sexual partners^a^				Casual sex		Group sex		Rare condom use^b^	
	Model 1 (age)		Model 2 (years since)							
Variable	aOR	95% CI	aOR	95% CI	aOR	95% CI	aOR	95% CI	aOR	95% CI
Age/years since sexual initiation^c^	1.04*	(1.01–1.07)	1.07***	(1.03–1.10)	0.99	(0.96–1.01)	1.01	(0.97–1.05)	1.00	(0.97–1.04)
Education level < college	1.00	(0.59–1.70)	0.92	(0.53–1.58)	1.01	(0.66–1.55)	0.91	(0.43–1.92)	3.90***	(2.25–6.76)
Employment (ref: Work-study/in school)										
Full-time job	2.19*	(1.16–4.16)	2.04*	(1.06–3.92)	1.38	(0.84–2.28)	1.67	(0.70–3.99)	0.96	(0.51–1.82)
Unemployed/part-time job/military	1.49	(0.61–3.65)	1.22	(0.47–3.14)	1.56	(0.76–3.19)	2.05	(0.65–6.45)	1.19	(0.53–2.65)
Living with marital or romantic partner	0.79	(0.43–1.42)	0.73	(0.40–1.31)	1.59*	(1.01–2.51)	1.48	(0.68–3.25)	0.91	(0.52–1.61)
Homosexual/bisexual experience	3.24**	(1.61–6.52)	3.22**	(1.55–6.69)	1.85*	(1.02–3.36)	3.11**	(1.52–6.39)	1.84	(0.89–3.81)
Gender × early sexual initiation (ref: Female, non-early sexual initiation)										
Male, non-early sexual initiation	7.72***	(3.89–15.34)	7.94***	(3.90–16.15)	3.66***	(2.27–5.90)	3.25*	(1.28–8.24)	0.60	(0.35–1.03)
Female, early sexual initiation	8.78**	(2.54–30.31)	8.14**	(2.41–27.48)	8.35***	(2.95–23.59)	6.58**	(1.62–26.68)	2.95	(0.99–8.77)
Male, early sexual initiation	12.63***	(5.08–31.41)	9.93***	(3.77–26.16)	5.24***	(2.55–10.75)	9.85***	(3.63–26.76)	0.57	(0.25–1.33)

^a^ ≥ 8 lifetime sexual partners, with adjustment for either age (model 1) or years since sexual initiation (model 2)

^b^ Including use frequency of “seldom” and “never”

^c^ Age was included in the model for casual sex, group sex, and rare condom use

* *p* < .05. ** *p* < .01. ***p < .001

Before undertaking the mediation analysis, we evaluated an alternative role of preceding-sex use of illicit drugs as a confounder on the relationship between early sexual initiation and risky sexual practice. After adding preceding-sex use of illicit drug as a covariate in the multivariable logistic regression model, reported in Additional file 1: Supplementary Table S1, the significance pattern of the dummy variables for the interaction between gender and early sexual initiation remained similar to that of Table 4, with modest changes in the magnitude of aOR for male and female early sexual initiators, respectively.

Treating preceding-sex use of illicit drugs as a mediator between early sexual initiation and risky sexual practices, we then ran a series of causal mediation analyses (Table 5). For the risky sexual practices that had a prevalence greater than 10%, including multiple sexual partners (17.9%), casual sex (36.1%), and rare condom use (22.2%), Poisson regression was used, whereas for group sex (7.0%) logistic regression was used for the outcome modeling. The direct effects, indirect effects, and total effects were significant for three of the risky sexual practices tested, including multiple sexual partners, casual sex, and group sex. The indirect effect proportion of the total effect was expressed as a percentage on a log scale and ranged from approximately 17% (group sex and multiple sexual partners) to 19% (casual sex), with a higher percentage implying that more of the total effect was mediated by preceding-sex use of illicit drugs.

**Table 5:** Causal mediation analysis examining illicit drug use right before sex as a potential mediator between early sexual initiation and risky sexual practices among alcohol- and tobacco-using young adults in Taipei metropolitan area recruited using RDS during 2007–2010 (*N* = 916)

Exposure (E)	Mediating variable (M)	Outcome variable (Y)	Direct effect	Indirect effect	Total effect	Indirect/Total (%)^a^
Early sexual initiation	Illicit drug use right before sex	Multiple sexual partners	1.61 (1.15–2.21)^b^	1.10 (1.03–1.23)^b^	1.77 (1.24–2.41)^b^	16.7
		Casual sex	1.35 (1.08–1.67)^b^	1.07 (1.03–1.15)^b^	1.44 (1.17–1.77)^b^	18.6
		Group sex	2.41 (1.23–4.87)^b^	1.19 (1.04–1.49)^b^	2.86 (1.46–5.37)^b^	16.6
		Rare condom use	1.33 (0.92–1.85)	0.99 (0.94–1.02)	1.31 (0.91–1.81)	−3.72

^a^ Calculated on the natural log-scale

^b^Statistically significant based on bootstrap 95% confidence intervals (in parenthesis)

(1) All models controlled for male gender, age, education < college, employment, living with marital or romantic partner, and homosexual/bisexual experience, (2) Poisson regression was used for the outcome regression model (E and M predicting Y) for multiple sexual partners, casual sex, and rare condom use due to nonrare outcomes (prevalence > 10%). Logistic regression was used for group sex (prevalence < 10%)

## Discussion

Among this RDS-recruited sample of alcohol- and tobacco-using young adults in the Taipei metropolitan area, early sexual initiators had a higher prevalence of regular binge drinking, illicit drug use, and risky sexual practices than nonearly sexual initiators, though the type of illicit drugs and risky sexual practices differed between males and females. After controlling for potential sociodemographic confounders, multivariable logistic regression analyses revealed that higher odds of multiple sexual partners, casual sex, and group sex were consistently associated with early sexual initiation, gender, and their interaction. Causal mediation analyses found that preceding-sex use of illicit drugs was a significant mediator for these risky sexual practices, with the proportions mediated ranging from 17 to 19%. These findings have implications for the prevention of risky sexual practice among alcohol- and tobacco-using young adults.

It is noteworthy that the contrast between female early sexual initiators and female nonearly sexual initiators seemed to be less disadvantaged in terms of education (less difference in educational level), employment (greater proportion holding a full-time job), and substance use (less increase in prevalences) than the contrast between their male counterparts. Since females in Taiwan typically have a much lower prevalence of tobacco smoking or problematic alcohol drinking than males [32], the criteria of alcohol- and tobacco-using might thus recruit a special subgroup of females with economic affordability for regular use of these two substances. Furthermore, female early sexual initiators were significantly younger than female nonearly sexual initiators, whereas male early sexual initiators were not different in age from male nonearly sexual initiators. All these ended up in ascertaining a group of female early sexual initiators who had better economic autonomy, which might explain their higher educational level and better employment status, and a more open attitude toward sexual autonomy. However, these observations are limited by the small number of female early sexual initiators and warrants further study to replicate these characteristics.

Under the context that all the participants in this study were regular users of alcohol and tobacco, the early sexual initiators still reported more regular binge drinking than the nonearly sexual initiators. This is consistent with previous studies showing early sexual initiators to have increased risk of binge drinking [57, 58]. Furthermore, the early sexual initiators reported more lifetime illicit drug use, in which ketamine was the most commonly used drug, and more preceding-sex use of illicit drugs than the nonearly sexual initiators. Although we did not inquire explicitly about the type of illicit drugs used in these occasions, their pattern of lifetime use of illicit drugs indicate that ketamine was likely to be the most commonly used illicit drug for the occasion. The associations of more frequent and extensive substance use with early sexual initiation among these alcohol- and tobacco-using young adults are consistent with the findings from cross-country comparisons [1, 34].

When the two groups of sexual initiation were compared for their risky sexual practices using the multivariable logistic regression, male early sexual initiators had greater aORs of multiple sexual partners and group sex than female early sexual initiators did, as compared to female nonearly sexual initiators. These findings, consistent with previous reports [3, 5, 59], might be attributed to the sexual double-standard in unequal acceptance of liberal sexual practices between genders that traditionally favors males [10, 60, 61]. It is noteworthy that two-thirds of our participants with group sex experience were heterosexual, which seems to exceed the expectation given the conservative East Asian collective culture [12, 13]. Since heterosexual group sex has not been discussed extensively in the literature [62], this non-negligible prevalence of group sex in the non-sexual-minority population calls for more future investigation. In contrast, female early sexual initiators had a greater aOR for casual sex than male early sexual initiators. This indicates that female young adults who used alcohol and tobacco regularly and had an early sexual initiation might be less willing to commit to a stable relationship than male counterparts. This warrants further investigation to examine whether this is due to openness toward liberal sexual practice or job concerns.

When the role of preceding-sex use of illicit drugs on the relationship between early sexual initiation and risky sexual practice is considered, there are three possibilities: a confounder, a collider, or a mediator [63]. To be a confounder would imply that preceding-sex use of illicit drugs is associated with the exposure (i.e., early sexual initiation) and is an independent cause for the outcome (i.e., risky sexual experience). However, adding it to the multivariable logistic model only led to modest changes in the aOR of the early sexual initiation on risky sexual practice. Given that repetitive preceding-sex use of illicit drugs presumably occurs following sexual initiation and empirical evidence suggests that the majority of Asian club drug users began their first club drug use after their sexual initiation [6], this pattern of attenuated aOR of the exposure after the adding of preceding-sex use of illicit drugs is compatible with its role as a mediator. Nevertheless, a mediator cannot be distinguished from a confounder solely based on the statistical change. Instead, preceding-sex use of illicit drugs was treated as a mediator in this study because of our postulation. To be a collider would imply that preceding-sex use of illicit drugs is a consequence of both early sexual initiation and risky sexual experience, which cannot be ruled out in this study. Hence, the results presented in Table 4 do not include preceding-sex use of illicit drugs as a covariate to avoid potential spurious association that may result from conditioning on a collider [64, 65]. Taken together, the most appropriate role of preceding-sex use of illicit drugs on the relationship between early sexual initiation and risky sexual practice is a mediator.

Our mediation analyses explored the preceding-sex use of illicit drugs as a possible mechanism behind the associations between early sexual initiation and risky sexual practices. Preceding-sex use of illicit drugs best explained the relationship between early sexual initiation and casual sex. There might be a common venue for both illicit drug use and casual sex, such as nightlife settings, where attendees might have opportunities to “get high” and solicit uncommitted sexual partners. Meanwhile, multiple sexual partners and group sex had slightly smaller proportions explained by the preceding-sex use of illicit drugs. Both sexual practices were less specific with regard to the settings under which sex had taken place and could not specify whether regular or uncommitted partners were involved. A mix of these factors might have contributed to a lower explanatory power of the preceding-sex use of illicit drugs.

Another possible factor underlying all the associations might be participants’ personality characteristics such as sensation seeking and impulsivity. As previous studies indicated, sensation seeking and impulsivity may mediate the early sexual initiation into illicit drug use and risky sexual practice [40–45]. Since our questionnaire did not measure these personality characteristics, we could not evaluate whether this pathway might also account for the mediation effect of the preceding-sex use of illicit drugs.

Intriguingly, early sexual initiation was not consistently associated with rare condom use, with the association existing only in females. This suggests that females were in a position of lower power than males in heterosexual relationships originating from a traditional view of females being subordinated. The complicated relationship between rare condom use and early sexual initiation is also reflected in the inconsistent findings in the literature. One study found that early sexual initiation was associated with inconsistent condom use [5], but another study found no correlation among women between early sexual initiation and negative condom attitudes [4].

### Implications

The current findings have implications for enhancing the sexual health of alcohol- and tobacco-using young adults. First, timely sexual health intervention should be tailored for this substance-using population (particularly among females), as they are more likely to have their first sexual encounter at a rather young age and to be involved in risky sexual practices. It is critical to help them establish a low-risk partnering pattern during their initial exploration of sexuality, one characterized by safe sex practices and effective sexual communication.

Second, public sexual health education should incorporate the prevention of binge drinking and illicit drug use as an additional major theme for the substance-using population. Both can undermine the capacity to use protection and to refrain from unwanted sexual solicitation. In particular, as early initiators were likely to have casual sex, it is critical to inform them of the potential sexual health risks incurred with binge drinking and illicit drug use.

### Limitations

There are limitations in this study. First, this study lacked the event-level information on risky sexual practices and right-before-sex use of illicit drugs. We did not know the context of participants’ sexual practice, e.g., reasons for casual sex, and therefore the designation of certain sexual practices as risky was based on averaged effect from the literature. In addition, we were not able to determine if illicit drugs were used right before a particular instance of risky sexual practice. The mediation effects should be interpreted as preliminary findings. Second, causal mediation analysis requires assumptions of no unmeasured confounding. This is difficult to evaluate with our data, but we have included all relevant variables as potential confounders in the mediation models. Lastly, this study conducted different types of statistical analyses that do not have clear guidance to adjust for multiple testing. Hence, our findings warrant future replication in other settings.

Future studies can turn to samples of young people recruited from nightclubs and sexually transmitted infection clinics, who are more likely to have experience of preceding-sex illicit drug use. To reduce potential bias in the interpretation of causal mediation, preceding-sex use of illicit drugs and risky sexual practices should be assessed on event-level [25], in addition to our lifetime measures. Lastly, to better define risky sexual practices, event-level unprotected sex should be adopted as the primary indicator.

## Conclusions

In summary, our study presented valuable evidence to demonstrate that early sexual initiation was consistently linked with various risky sexual practices in an East Asian context, with preceding-sex use of illicit drugs mediating part of the association. Under a traditionally conservative culture with evolving sexual attitudes, early sexual initiation has important implications for sexual behavior patterns and sexual health. In particular, the young substance-using population is at risk and deserves additional public health attention.

## Supplementary Information

**Additional file 1: Supplementary Methods. Supplementary Results. Table S1.** Weighted multivariable logistic regression models of multiple sexual partners, casual sex, group sex, and rare condom use on early sexual initiation among alcohol- and tobacco-using young adults in Taipei metropolitan area recruited using RDS during 2007–2010 (*N* = 916). **Table S2.** Condom use (in original options) among sexually experienced alcohol- and tobacco-using young adults in Taiwan recruited using RDS during 2007–2010, by gender and sexual initiation (*N* = 916). **Table S3.** Relations of sexual partners and relationship status to condom use (in original options) among sexually experienced alcohol- and tobacco-using young adults in Taiwan recruited using RDS during 2007–2010, by gender (*N* = 916). **Table S4.** Relations of sexual partners and relationship status to condom use (in original options) among sexually experienced alcohol- and tobacco-using young adults in Taiwan recruited using RDS during 2007–2010, by gender and early sexual initiation (*N* = 916). **Table S5.** Weighted multivariable logistic regression analysis of rare condom use on early sexual initiation among alcohol- and tobacco-using young adults in Taiwan recruited using RDS during 2007–2010 (*N* = 916).

**Additional file 2.** Questionnaire for the variables used in the study.

## Data Availability

The authors received permission to access the data used in this study; however, they are unable to share the data as they are not the data custodian. Data access queries can be directed to Division of Controlled Drugs, Taiwan Food and Drug Administration, Ministry of Health and Welfare, Executive Yuan, Taipei, Taiwan.

## Abbreviations

ACASI: Audio computer-assisted self-interview
aOR: Adjusted odds ratio
FM2: Flunitrazepam
GHB: Gamma hydroxybutyrate
OR: Odds ratio
RDS: Respondent-driven sampling
