# Supplementary material for: Early sexual initiation and risky sexual practices among alcohol- and tobacco-using young adults in Taiwan: mediation analysis of preceding-sex use of illicit drugs
Source: BMC Public Health. 2020 Nov 3;20:1647. doi: 10.1186/s12889-020-09777-0 (PMC7641850; doi:10.1186/s12889-020-09777-0)
Supplement: Supplementary file 1 — Additional file 1: Supplementary Methods. Supplementary Results. Table S1. Weighted multivariable logistic regression models of multiple sexual partners, casual sex, group sex, and rare condom use on early sexual initiation among alcohol- and tobacco-using young adults in Taipei metropolitan area recruited using RDS during 2007–2010 (N = 916). Table S2. Condom use (in original options) among sexually experienced alcohol- and tobacco-using young adults in Taiwan recruited using RDS during 2007–2010, by gender and sexual initiation (N = 916). Table S3. Relations of sexual partners and relationship status to condom use (in original options) among sexually experienced alcohol- and tobacco-using young adults in Taiwan recruited using RDS during 2007–2010, by gender (N = 916). Table S4. Relations of sexual partners and relationship status to condom use (in original options) among sexually experienced alcohol- and tobacco-using young adults in Taiwan recruited using RDS during 2007–2010, by gender and early sexual initiation (N = 916). Table S5. Weighted multivariable logistic regression analysis of rare condom use on early sexual initiation among alcohol- and tobacco-using young adults in Taiwan recruited using RDS during 2007–2010 (N = 916). [file 12889_2020_9777_MOESM1_ESM.doc]

**Additional file 1**

**Content:**

Supplementary Methods.

Supplementary Results.

Table S1. Weighted multivariable logistic regression models of multiple sexual partners, casual sex, group sex, and rare condom use on early sexual initiation among alcohol- and tobacco-using young adults in Taipei metropolitan area recruited using RDS during 2007-2010 (*N* = 916).

Table S2. Condom use (in original options) among sexually experienced alcohol- and tobacco-using young adults in Taiwan recruited using RDS during 2007-2010, by gender and sexual initiation (*N* = 916)

Table S3. Relations of sexual partners and relationship status to condom use (in original options) among sexually experienced alcohol- and tobacco-using young adults in Taiwan recruited using RDS during 2007-2010, by gender (*N* = 916)

Table S4. Relations of sexual partners and relationship status to condom use (in original options) among sexually experienced alcohol- and tobacco-using young adults in Taiwan recruited using RDS during 2007-2010, by gender and early sexual initiation (*N* = 916)

Table S5. Weighted multivariable logistic regression analysis of rare condom use on early sexual initiation among alcohol- and tobacco-using young adults in Taiwan recruited using RDS during 2007-2010 (*N* = 916)

**Supplementary Methods**

*1. Recruitment of seeds*

The study recruited 47 seeds from either community-based or hospital-based settings. Among 35 seeds from the community sites, their sources included young smokers who drank beer in nightclubs, frequent KTV party (i.e., individual-room karaoke bar) attendees who used alcohol and tobacco, web forum users who shared information on nightlife and drinking activities, college students whose nightlife affected school performance and were referred by college counselors. The remaining 12 seeds were recruited from substance misuse patients referred by physicians at rehabilitation centers. Seeds and eligible participants could invite up to six peers who satisfied the sampling criteria to participate in the study.

*2. Balancing the need for referral and confidentiality*

The research team undertook several procedures to balance the need for referral and confidentiality (Ting et al., 2015). For example, each participant was asked about his or her nickname and preferred way of communication (mostly mobile phone number). Then all recruits were asked about the nickname of their recruiters and their relationship to verify and subsequently delineate their network structures. Afterward, participants were asked to sign an informed consent with a nickname.

To balance the assurance of anonymity in their participation in this study and the research team’s communication during the recruitment process, all recruits were asked for nicknames and mobile phone numbers of their recruiters and their relationships to verify and subsequently delineate their network structures. We were able to use this information to match recruits with their recruiters efficiently, similar to the use of coupons in the RDS literature but without compromising privacy. In addition, we asked all participants to estimate the number of their peers who satisfied the two sampling criteria, as in other RDS studies, for the purpose of weighting in the RDS estimation.

*3. Participants’ alcohol and tobacco use*

When RDS recruitment was carried out, part of the criteria was regular use of tobacco and alcohol; but it was not explicitly defined to allow for easier recruitment of participants. Based on participants’ response to the interview, 84.3% consumed alcohol in the past-1-year (26.6% being ≥ 1 day per week, 17.2% being 2-4 days per month, and 40.5% being ≤ 1 day per month). Regarding their tobacco use, 83.2% smoked tobacco in the past-1-year (63.3% being almost daily, 10% being 1-4 days per week, and 9.9% being ≤ 2-4 days per month and ≥ 1 day in the past 12 months). For comparison, in the 2014 National Survey of Substance Use among people aged 12 to 64 years, the prevalence of past-1-year use was 48.48% for alcohol drinking and 19.73% for tobacco smoking (Chen et al., 2017).

*4. Questionnaire content*

**Demographics.** Participants self-reported gender, age, highest education completed, and current employment status. Regarding participants’ relationship status, a respondent was coded as living with marital or romantic partner by his or her answer to the question “*who are you living with?*” (*parents, relatives, friends, alone, husband/wife, boyfriend/girlfriend, others*) as “*husband/wife*” or “*boyfriend/girlfriend*”.

**Sexual experience.** We defined sexually experienced as having experience in any of the following three forms: vaginal sex, oral sex, or anal sex. In the survey, these terms were written out exactly when the participants were asked if they had experience in these types of sexual behaviors. Since various types of sexually transmitted infections could be spread through oral sex, we counted it as sexual experience. Based on information about past engagement and the age at first engagement in vaginal sex, anal sex, and oral sex, respectively, we categorized a respondent as an early sexual initiator if the age reported for any one type of sexual behaviors was lower than 16. Four types of risky sexual practices were further defined as follows: (1) multiple sexual partners: the number of lifetime sexual partners was measured in categorical form (*1, 2-3, 4-5, 6-7, 8-10, 11-14, 15 or above*). Based on its distribution in the corresponding order among the 915 participants (23.8%, 28.9%, 18.1%, 11.4%, 7.0%, 2.5%, and 8.4%), we created a variable “multiple sexual partner” to reflect participants who had ≥ 8 partners in lifetime given the prevalence above that was less than one-fifth; (2) casual sex: such experience was inquired as *“Did you ever had sex with people whom you had just met, unfamiliar people, or strangers, so-called one-night stand?”* (*never, once, 2-3 times, 4-5 times, 6-9 times, 10 times or above*) and was recoded as yes to a binary variable if a respondent ever had such experience; (3) group sex: such experience was inquired as *“Did you ever have sex with two or more people at the same time or in a row (gang bang)?”* (*never, once, 2-3 times, 4-5 times, 6-9 times, 10 times or above*) and was recoded as yes to a binary variable if a respondent ever had such experience; and (4) rare condom use: experience of condom use was inquired as “*In general, what is your frequency of using condoms throughout sex?*” (*always, often, sometimes, seldom, never*). A condom use frequency of “seldom” or “never” was recoded as yes for rare condom use.

Two variables related to sexual experiences were defined as follows: (1) preceding-sex use of illicit drugs: for respondents who reported lifetime use of any illicit drugs, they were further questioned about their experience of using such drugs right before sex: “The number of times you used such drugs right before sex?” We dichotomized the original answers (never, once, 2-3 times, 4-5 times, 6-9 times, 10 times or above) into a yes or no format; (2) homosexual/bisexual experience: for respondents with sexual experience, they were further inquired about “*What gender(s) of people have you had sex with so far?*” (*male, female, both genders*). We used the information regarding the gender(s) of people with whom the respondent had sex (male, female, both genders) to define homosexual/bisexual experiences.

**Substance/drug use experience.** Use of licit substances (tobacco, alcohol, and areca nut) and illicit drugs was assessed by asking participants a series of questions worded as follows: “Have you ever, even once, used [licit substance or illicit drug name]?” For each substance/drug endorsed, respondents were asked further substance/drug-specific questions regarding age of first use, situation of first use, average frequency of consumption, and recency of use. Alcohol use was defined in our questionnaire as consuming a cup of alcoholic drink (e.g., about 100 c.c. beer) at least once. For this study, variables on substance/drug use included the following: (1) regular binge drinking: alcohol use experience was inquired as “*When you drink alcohol,* h*ow often do you drink 5 or more “alcohol units” in a row?*” (*every time, almost every time, sometimes, once in a while, just once, never*). This item was accompanied by pictures and text to explain the amount of one alcohol unit. Based on its distribution in the corresponding order among the 915 participants (5.3%, 10.6%, 12.7%, 22.2%, 1.1%, and 48.1%), we recoded the item in a binary variable by collapsing every time and almost every time into one category to reflect a general tendency of binge drinking; and (2) hard drug vs. club drug use: hard drugs here referred to heroin and methamphetamine, which are classified as Schedule I and II drugs, respectively, in Taiwan, while club drugs referred to ecstasy, ketamine, cannabis, FM2 (flunitrazepam), angel dust (phencyclidine), and GHB (4-hydroxybutanoic acid), which are classified as Schedule III drugs in Taiwan except cannabis and ecstasy (classified as Schedule II). Since hard drug and club drug users had different demographic and sexual partnership profiles (Chen et al., 2017), we distinguished between them using two additional variables. First, we created a variable “hard drug use” to represent participants who ever used heroin or methamphetamine, regardless of involvement with other types of illicit drugs. Second, we also created a variable “exclusively club drug use” to represent participants who only used club drugs, such as ecstasy, marijuana, and ketamine, but not hard drugs. The two variables together represented all illicit drug users in this survey.

**Reliability and validity of the questionnaire.** This study’s items on substance use and development-related behaviors were derived from a web-based questionnaire for adolescent substance use, in which a two-week test-retest reliability study among 67 junior-high school students showed excellent kappas for the items on the use of readily available substances (0.81 for alcohol drinking, 0.93 for tobacco smoking, and 1.0 for betel nut chewing) and the percent of agreement was close to 100% for the items on illicit drugs and development-related behaviors (Wang et al., 2005). Although self-report of illicit drug use had been found to have a low sensitivity, around 30% for amphetamines or opioids among patients at emergency department in Taiwan (Chen et al., 2006) and similar figures in other epidemiological surveys (Fendrich et al., 1999; Colon et al., 2001), a randomized trial did reveal that the privacy-enhancing technology such as web-based questionnaire led to a greater reporting rate of deviant behavior such as truancy, alcohol drinking, tobacco smoking, and amphetamine use compared to the traditional mode of paper-and-pencil questionnaire administration (Wang et al., 2005).

**References cited in supplementary methods:**

Chen, W. J., Fang, C.-C., Shyu, R.-S., Lin, K.-C. 2006. Underreporting of illicit drug use by patients at emergency departments as revealed by two-tiered urinalysis. Addict Behav*,* 31, 2304-2308.

Chen, W. J., Wu, S.-C., Tsay, W.-I., Chen, Y.-T., Hsiao, P.-C., Yu, Y.-H., Ting, T.-T., Chen, C.-Y., Tu, Y.-K., Huang, J.-H., Yang, H.-J., Li, C.-Y., Strong, C., Yen, C.-F., Yen, C.-F., Hsu, J. 2017. Differences in prevalence, socio-behavioral correlates, and psychosocial distress between club drug and hard drug use in Taiwan: Results from the 2014 National Survey of Substance Use. Int J Drug Policy*,* 48, 99-107.

Colon, H. M., Robles, R. R., Sahai, H. 2001. The validity of drug use responses in a household survey in Puerto Rico: comparison of survey responses of cocaine and heroin use with hair tests. Int J Epidemiol*,* 30, 1042-1049.

Fendrich, M., Johnson, T. P., Sudman, S., Wislar, J. S., Spiehler, V. 1999. Validity of drug use reporting in a high-risk community sample: a comparison of cocaine and heroin survey reports with hair tests. Am J Epidemiol*,* 149, 955-962.

Ting, T.-T., Chen, C.-Y., Tsai, Y.-S., Chen, Y.-T., Su, L.-W., Chen, W. J. 2015. Using social network as a recruiting tool for research on substance use in the Taipei metropolitan area: Study design, implementation, and epidemiological estimates. J Epidemiol*,* 25, 647-655.

Wang, Y.-C., Lee, C.-M., Lew-Ting, C.-Y., Hsiao, C. K., Chen, D.-R., Chen, W. J. 2005. Survey of substance use among high school students in Taipei: Web-based questionnaire versus paper-and-pencil questionnaire. J Adolesc Health*,* 37, 289-295.

**Supplementary Results**

*Early sexual initiation and rare condom use*

To further explore the lack of association of early sexual initiation with rare condom use, we first examined the distribution of condom use in its original options and found only female early sexual initiators had higher proportions in the frequency of seldom or never than female nonearly sexual initiators (Supplementary **Table S2**). Then we examined the relations of the number of sexual partner to condom use in its original options (Supplementary **Table S3**). For participants with one sexual partner in lifetime, their condom use frequencies were either the most or the least frequent. As the number of sexual partners increased, participants’ condom use frequency gradually clustered towards the center. This might imply that participants with only one partner were either the most cautious (of pregnancy or sexually transmitted infections) or the most trusting of their only partner. People who had two or more partners tended to use condoms occasionally. Then we examined the relations of living with marital or romantic partner to condom use among early sexual initiators (Supplementary **Table S4**). Female early initiators who were not living with a steady partner were much more likely to report condom non-use than their male counterparts, implying the former being exposed to higher risks than the latter. If we add the number of sexual partners into the multiple variable logistic regression analysis (Supplementary **Table S5**), female early sexual initiators were positively associated with rare condom use, while having 2 to 5 lifetime sexual partners was negatively associated with rare condom use.

Table S1. Weighted multivariable logistic regression models of multiple sexual partners, casual sex, group sex, and rare condom use on early sexual initiation among alcohol- and tobacco-using young adults in Taipei metropolitan area recruited using RDS during 2007-2010 (*N* = 916).

|  | Multiple sexual partners^a^ | | | | |  | Casual sex | |  | Group sex | |  | Rare condom use^b^ | |
| --- | --- | --- | --- | --- | --- | --- | --- | --- | --- | --- | --- | --- | --- | --- |
|  | Model 1 (age) | |  | Model 2 (years since) | |  |  |  |  |  |  |  |  |  |
| Variable | aOR | 95% CI |  | aOR | 95% CI |  | aOR | 95% CI |  | aOR | 95% CI |  | aOR | 95% CI |
| Age/years since sexual initiation^c^ | 1.04* | (1.01-1.07) |  | 1.07*** | (1.03-1.11) |  | 0.99 | (0.96-1.01) |  | 1.01 | (0.97-1.05) |  | 1.00 | (0.97-1.04) |
| Education level < college | 0.92 | (0.53-1.58) |  | 0.84 | (0.48-1.47) |  | 0.94 | (0.61-1.44) |  | 0.86 | (0.40-1.84) |  | 3.87*** | (2.23-6.72) |
| Employment (ref: Work-study/in school) |  |  |  |  |  |  |  |  |  |  |  |  |  |  |
| Full-time job | 2.44** | (1.29-4.61) |  | 2.28* | (1.19-4.37) |  | 1.53 | (0.92-2.55) |  | 1.79 | (0.73-4.37) |  | 0.97 | (0.51-1.83) |
| Unemployed/part-time job/military | 1.50 | (0.60-3.74) |  | 1.25 | (0.49-3.23) |  | 1.48 | (0.76-2.92) |  | 2.06 | (0.64-6.62) |  | 1.17 | (0.52-2.63) |
| Living with marital or romantic partner | 0.76 | (0.42-1.37) |  | 0.71 | (0.39-1.27) |  | 1.59* | (1.00-2.53) |  | 1.43 | (0.64-3.18) |  | 0.91 | (0.52-1.61) |
| Homosexual/bisexual experience | 3.09** | (1.58-6.05) |  | 3.07** | (1.52-6.17) |  | 1.75 | (0.97-3.17) |  | 2.99** | (1.49-6.01) |  | 1.82 | (0.87-3.8) |
| Preceding-sex illicit drug use | 2.96** | (1.38-6.35) |  | 2.87** | (1.30-6.31) |  | 4.65** | (1.90-11.43) |  | 1.99 | (0.86-4.63) |  | 1.22 | (0.51-2.95) |
| Gender × early sexual initiation  (ref: Female, non-early sexual initiation) |  |  |  |  |  |  |  |  |  |  |  |  |  |  |
| Male, non-early sexual initiation | 7.69*** | (3.86-15.30) |  | 7.85*** | (3.87-15.94) |  | 3.81*** | (2.39-6.07) |  | 3.24* | (1.27-8.25) |  | 0.60 | (0.35-1.03) |
| Female, early sexual initiation | 9.18** | (2.59-32.49) |  | 8.43** | (2.45-28.99) |  | 9.22*** | (3.23-26.28) |  | 6.77** | (1.68-27.28) |  | 2.97 | (0.99-8.88) |
| Male, early sexual initiation | 10.29*** | (4.24-24.94) |  | 8.06*** | (3.15-20.65) |  | 4.27*** | (2.11-8.63) |  | 8.45*** | (3.10-23.02) |  | 0.55 | (0.23-1.29) |

^a^ ≥ 8 lifetime sexual partners, with adjustment for either age (model 1) or years since sexual initiation (model 2).

^b^ Including use frequency of “seldom” and “never”

^c^ Age was included in the model for casual sex, group sex, and rare condom use.

* p<.05. ** p<.01. ***p<.001

Table S2. Condom use (in original options) among sexually experienced alcohol- and tobacco-using young adults in Taipei metropolitan area recruited using RDS during 2007-2010, by gender and sexual initiation (*N* = 916)

|  | Nonearly sexual initiation | | |  | Early sexual initiation | | |  |  |
| --- | --- | --- | --- | --- | --- | --- | --- | --- | --- |
|  | *N* | %_wt_ | 95% CI |  | *N* | %_wt_ | 95% CI |  | *p*^a^ |
| *Males* | *N* = 499 | | |  | *N* = 79 | | |  |  |
| Condom use |  |  |  |  |  |  |  |  | .518 |
| Every time | 144 | 28.9 | (23.4-35.7) |  | 19 | 19.3 | (9.5-28.9) |  |  |
| Often | 161 | 32.3 | (26.6-38.6) |  | 21 | 29.2 | (15.8-42) |  |  |
| Sometimes | 102 | 18.3 | (14.2-23.1) |  | 18 | 24.6 | (11.4-40.9) |  |  |
| Seldom | 67 | 12.9 | (8.8-16.9) |  | 14 | 15.4 | (5.8-27.3) |  |  |
| Never | 25 | 7.6 | (3.5-11) |  | 7 | 11.6 | (3-23.7) |  |  |
|  |  |  |  |  |  |  |  |  |  |
| *Females* | *N* = 308 | | |  | *N* = 30 | | |  |  |
| Condom use |  |  |  |  |  |  |  |  | .048* |
| Every time | 73 | 23.2 | (16.1-31.6) |  | 3 | 7.8 | (0.0-19.9) |  |  |
| Often | 75 | 20.5 | (14.7-28.3) |  | 7 | 15.3 | (2.5-38.1) |  |  |
| Sometimes | 68 | 21.9 | (15.9-29.9) |  | 7 | 11.6 | (0.6-24.9) |  |  |
| Seldom | 58 | 16.8 | (11.4-24.3) |  | 10 | 45.5 | (18-70.4) |  |  |
| Never | 34 | 17.5 | (7.9-25.1) |  | 3 | 19.8 | (0-44.4) |  |  |

^a^Using weighted chi-square tests, with weight exported from RDSAT; * p < 0.05

Table S3. Relations of sexual partners and relationship status to condom use (in original options) among sexually experienced alcohol- and tobacco-using young adults in Taipei metropolitan area recruited using RDS during 2007-2010, by gender (*N* = 916)

|  |  |  | Condom use | | | | |  |
| --- | --- | --- | --- | --- | --- | --- | --- | --- |
|  |  |  | Every  time | Often | Sometimes | Seldom | Never |  |
| Variable | *N* |  | Weighted row % | | | | | p^a^ |
| *Male* |  |  |  |  |  |  |  |  |
| Number of sexual partners |  |  |  |  |  |  |  | <.001*** |
| 1 | 114 |  | 37.9 | 19.4 | 12.0 | 7.4 | 23.3 |  |
| 2-5 | 246 |  | 32.6 | 36.6 | 16.4 | 10.6 | 3.8 |  |
| 6 or more | 218 |  | 16.7 | 33.5 | 25.3 | 20.0 | 4.5 |  |
| Living with marital or romantic partner |  |  |  |  |  |  |  | .155 |
| Yes | 475 |  | 16.6 | 34.3 | 23.0 | 20.4 | 5.7 |  |
| No | 103 |  | 29.4 | 32.4 | 18.2 | 11.8 | 8.2 |  |
|  |  |  |  |  |  |  |  |  |
| *Female* |  |  |  |  |  |  |  |  |
| Number of sexual partners |  |  |  |  |  |  |  | .102 |
| 1 | 78 |  | 24.4 | 13.8 | 17.2 | 17.0 | 27.6 |  |
| 2-5 | 187 |  | 26.2 | 19.0 | 23.2 | 15.7 | 15.9 |  |
| 6 or more | 338 |  | 8.0 | 32.9 | 20.3 | 32.9 | 5.9 |  |
| Living with marital or romantic partner |  |  |  |  |  |  |  | .209 |
| Yes | 229 |  | 22.0 | 19.9 | 22.2 | 15.3 | 20.6 |  |
| No | 109 |  | 22.0 | 22.2 | 20.1 | 27.1 | 8.7 |  |

^a^Using weighted chi-square tests, with weight exported from RDSAT; *** p < 0.001

Table S4. Relations of sexual partners and relationship status to condom use (in original options) among sexually experienced alcohol- and tobacco-using young adults in Taipei metropolitan area recruited using RDS during 2007-2010, by gender and early sexual initiation (*N* = 916)

|  |  |  | Condom use | | | | |  |
| --- | --- | --- | --- | --- | --- | --- | --- | --- |
|  |  |  | Every time | Often | Sometimes | Seldom | Never |  |
| Variable | *N* |  | Weighted row % | | | | | p^a^ |
| *Male early initiators* |  |  |  | | | | |  |
| Number of sexual partners |  |  |  |  |  |  |  | .002** |
| 1 | 8 |  | 74.9 | 12.9 | 11.6 | 0.0 | 0.5 |  |
| 2-5 | 27 |  | 6.5 | 49.0 | 30.2 | 6.4 | 7.9 |  |
| 6 or more | 44 |  | 16.2 | 17.2 | 26.2 | 23.9 | 16.5 |  |
| Living with marital or romantic partner |  |  |  |  |  |  |  | .010* |
| Yes | 65 |  | 12.8 | 27.7 | 29.5 | 15.9 | 14.1 |  |
| No | 14 |  | 46.6 | 39.6 | 5.6 | 7.8 | 0.4 |  |
| *Male nonearly initiators* |  |  |  | | | | |  |
| Number of sexual partners |  |  |  |  |  |  |  | <.001*** |
| 1 | 106 |  | 35.4 | 19.8 | 12.1 | 7.9 | 24.8 |  |
| 2-5 | 219 |  | 36.0 | 34.6 | 14.9 | 11.0 | 3.4 |  |
| 6 or more | 174 |  | 16.8 | 36.8 | 25.1 | 19.2 | 2.1 |  |
| Living with marital or romantic partner |  |  |  |  |  |  |  |  |
| Yes | 410 |  | 31.9 | 32.8 | 16.7 | 11.3 | 7.4 | .040* |
| No | 89 |  | 13.3 | 33.9 | 25.1 | 21.5 | 6.3 |  |
|  |  |  |  |  |  |  |  |  |
| *Female early initiators* |  |  |  |  |  |  |  |  |
| Number of sexual partners |  |  |  |  |  |  |  | .032* |
| 1 | 2 |  | 0.0 | 0.0 | 0.0 | 100.0 | 0.0 |  |
| 2-5 | 12 |  | 22.0 | 11.0 | 15.6 | 6.9 | 44.5 |  |
| 6 or more | 16 |  | 0.0 | 19.3 | 13.5 | 67.2 | 0.0 |  |
| Living with marital or romantic partner |  |  |  |  |  |  |  | .981 |
| Yes | 17 |  | 11.5 | 14.9 | 13.9 | 46.6 | 13.0 |  |
| No | 13 |  | 6.4 | 14.9 | 18.1 | 41.0 | 19.6 |  |
| *Female nonearly initiators* |  |  |  |  |  |  |  |  |
| Number of sexual partners |  |  |  |  |  |  |  | .241 |
| 1 | 76 |  | 24.7 | 14.0 | 17.4 | 16.1 | 27.9 |  |
| 2-5 | 175 |  | 26.5 | 19.3 | 23.6 | 16.2 | 14.3 |  |
| 6 or more | 57 |  | 10.0 | 36.4 | 22.0 | 24.1 | 7.4 |  |
| Living with marital or romantic partner |  |  |  |  |  |  |  | .147 |
| Yes | 212 |  | 22.4 | 20.0 | 22.6 | 14.2 | 20.8 |  |
| No | 96 |  | 25.7 | 23.9 | 20.5 | 23.7 | 6.1 |  |

^a^Using weighted chi-square tests, with weight exported from RDSAT; * p < 0.05, ** p < 0.01, *** p < .001

Table S5. Weighted multivariable logistic regression analysis of rare condom use on early sexual initiation among alcohol- and tobacco-using young adults in Taipei metropolitan area recruited using RDS during 2007-2010 (*N* = 916)

|  |  | Rare condom use^b^ | |
| --- | --- | --- | --- |
|  |  |  |  |
| Variable |  | aOR | 95% CI |
| Age |  | 1.00 | (0.96-1.04) |
| Education level < college |  | 3.86*** | (2.24-6.66) |
| Employment (ref: Work-study/in school) |  |  |  |
| Full-time job |  | 1.06 | (0.57-1.96) |
| Unemployed/part-time job/military |  | 1.31 | (0.58-2.94) |
| Living with marital or romantic partner |  | 0.96 | (0.55-1.69) |
| Homosexual/bisexual experience |  | 1.93 | (0.94-3.97) |
| Number of sexual partners (lifetime) (ref: 1) |  |  |  |
| 2-5 |  | 0.47* | (0.23-0.93) |
| 6 or more |  | 0.59 | (0.29-1.22) |
| Gender × early sexual initiation  (ref: Female, non-early sexual initiation) |  |  |  |
| Male, non-early sexual initiation |  | 0.62 | (0.37-1.05) |
| Female, early sexual initiation |  | 3.27* | (1.06-10.09) |
| Male, early sexual initiation |  | 0.62 | (0.27-1.43) |

^a^ Including use frequency of “seldom” and “never”

* p<.05. ***p<.001
