# Supplementary material for: Early sexual initiation and risky sexual practices among alcohol- and tobacco-using young adults in Taiwan: mediation analysis of preceding-sex use of illicit drugs
Source: BMC Public Health. 2020 Nov 3;20:1647. doi: 10.1186/s12889-020-09777-0 (PMC7641850; doi:10.1186/s12889-020-09777-0)
Supplement: Supplementary file 2 — Additional file 2. Questionnaire for the variables used in the study. [file 12889_2020_9777_MOESM2_ESM.docx]

**Questionnaire for the variables used in the study**

“Early sexual initiation and risky sexual practices among alcohol- and tobacco-using young adults in Taiwan: mediation analysis of preceding-sex use of illicit drugs”

Tat Leong Wu, Te-Tien Ting, Chuan-Yu Chen, Lien-Wen Su, Wei J. Chen

| **A. Sociodemographics** |  |
| --- | --- |
| 1. When is your birthdate? | (year) (month) (day) |
| 2. When is your gender? | 1. ( ) male  2. ( ) female |
| 3. What is the educational level of your parents? |  |
| a. father: | 1. ( ) primary school or less  2. ( ) junior high school  3. ( ) senior/vocational high school  4. ( ) junior college  5. ( ) college/university or above  6. ( ) not know |
| b. mother: | 1. ( ) primary school or less  2. ( ) junior high school  3. ( ) senior/vocational high school  4. ( ) junior college  5. ( ) college/university or above  6. ( ) not know |
| 4. Who are you living with? | 1. ( ) parents  2. ( ) relatives  3. ( ) friends  4. ( ) alone  5. ( ) husband/wife  6. ( ) boyfriend/girlfriend  7. ( ) others |

| 5. What is your educational level? | 1. ( ) primary school or less  2. ( ) junior high school  3. ( ) senior/vocational high school  4. ( ) junior college  5. ( ) college/university or above |
| --- | --- |
| 6. Are you in school or employed? | 1. ( ) a full-time student  2. ( ) a full-time employment  3. ( ) a part-time job  4. ( ) a student with a part-time job  5. ( ) in military service  6. ( ) none of above |

| **B. Sexual experience** |  |
| --- | --- |
| 1. Have you ever had sexual experience? |  |
| a. vaginal sex: | 1. ( ) Yes 2. ( ) No |
| b. anal sex: | 1. ( ) Yes 2. ( ) No |
| c. oral sex: | 1. ( ) Yes 2. ( ) No |
| 2. When did you have sexual experience for the first time |  |
| a. vaginal sex: | ( ) years old |
| b. anal sex | ( ) years old |
| c. oral sex | ( ) years old |
|  |  |
| 3. When you had your first sexual experience, how old was your sexual partner? (including vaginal sex, anal sex, or oral sex) | ( ) years old |
| 4. What gender(s) of people have you had sex with so far? | 1. ( ) male  2. ( ) female  3. ( ) both genders |
| 5. What is the number of your sexual partners so far? | 1. ( ) 1 person  2. ( ) 2-3 persons  3. ( ) 4-5 persons  4. ( ) 6-7 persons  5. ( ) 8-10 persons  6. ( ) 11-14 persons  7. ( ) 15 or more persons |
| 6. Did you ever have sex with two or more people at the same time or in a row (gang bang)? | 1. ( ) never  2. ( ) once  3. ( ) 2-3 times  4. ( ) 4-5 times  5. ( ) 6-9 times  6. ( ) 10 times or above |

| 7. Did you ever had sex with people whom you had just met, unfamiliar people, or strangers, so-called one-night stand? | 1. ( ) never  2. ( ) once  3. ( ) 2-3 times  4. ( ) 4-5 times  5. ( ) 6-9 times  6. ( ) 10 times or above |
| --- | --- |
| 8. What is the number of times you used illicit drugs right before sex? | 1. ( ) never  2. ( ) once  3. ( ) 2-3 times  4. ( ) 4-5 times  5. ( ) 6-9 times  6. ( ) 10 times or above |
| 9. What is the number of times you drank alcohol right before sex? | 1. ( ) never  2. ( ) once  3. ( ) 2-3 times  4. ( ) 4-5 times  5. ( ) 6-9 times  6. ( ) 10 times or above |
| 10. In general, what is your frequency of using condoms throughout sex? | 1. ( ) always  2. ( ) often  3. ( ) sometimes  4. ( ) seldom  5. ( ) never |

| **C. Substance use** |  |
| --- | --- |
| 1. When you drink alcohol, how often do you drink 5 or more “alcohol units” in a row? | 1. ( ) every time  2. ( ) almost every time  3. ( ) sometimes  4. ( ) once in a while  5. ( ) just once  6. ( ) never |
| 2. Have you ever, even once, used any illicit drugs/inhalants listed in the following? (multiple options) | 1. ( ) ecstasy  2. ( ) sniffing glue  3. ( ) amphetamines  4. ( ) ketamine  5. ( ) FM2  6. ( ) marijuana  7. ( ) heroin  8. ( ) angel dust (PCP)  9. ( ) GHB  10. ( ) sedatives/hypnotics without prescription  11. ( ) diet pills without prescription  12. ( ) steroids without prescription  13. ( ) other drugs:  14. ( ) none of above |
